# Development of a Novel Cell Surface Attachment System to Display Multi-Protein Complex Using the Cohesin-Dockerin Binding Pair

**DOI:** 10.4014/jmb.2105.05022

**Published:** 2021-07-06

**Authors:** Hyeok-Jin Ko, Heesang Song, In-Geol Choi

**Affiliations:** 1 Food Biotech R&D Center, Samyang Corp., Seongnam 13488, Republic of Korea; 2 Department of Biochemistry and Molecular Biology, Chosun University School of Medicine, Gwangju 61452, Republic of Korea; 3 Department of Biotechnology, College of Life Sciences and Biotechnology, Korea University, Seoul 02841, Republic of Korea

**Keywords:** *Escherichia coli* cell surface attachment, non-covalent interaction module, cohesin–dockerin, α-neoagarobiose hydrolase

## Abstract

Autodisplay of a multimeric protein complex on a cell surface is limited by intrinsic factors such as the types and orientations of anchor modules. Moreover, improper folding of proteins to be displayed often hinders functional cell surface display. While overcoming these drawbacks, we ultimately extended the applicability of the autodisplay platform to the display of a protein complex. We designed and constructed a cell surface attachment (CSA) system that uses a noncovalent protein–protein interaction. We employed the high-affinity interaction mediated by an orthogonal cohesin-dockerin (Coh-Doc) pair from *Archaeoglobus fulgidus* to build the CSA system. Then, we validated the orthogonal Coh-Doc binding by attaching a monomeric red fluorescent protein to the cell surface. In addition, we evaluated the functional anchoring of proteins fused with the Doc module to the autodisplayed Coh module on the surface of *Escherichia coli*. The designed CSA system was applied to create a functional attachment of dimeric α-neoagarobiose hydrolase to the surface of *E. coli* cells.

## Introduction

Microbial cell surface display (CSD) is regarded as a powerful tool in various biotechnological applications [1]. Many techniques have been developed using an assortment of anchoring proteins, such as outer membrane proteins, lipoproteins, subunits of surface appendages (*e.g.*, flagella and pilus), or S-layer proteins to display proteins of interest (POIs) on the surfaces of bacteria [2]. However, many critical factors can limit the functional and active display of POIs. For instance, the size and structure of POIs to be displayed can cause low-level display at the cell surface and their overexpression can lead to host cell toxicity [3]. Among many approaches undertaken to overcome these limitations, “autodisplay” using autotransporters as the anchoring scaffold has been one of the most promising techniques because of lower levels of cellular toxicity and the ability to incorporate POIs of various sizes [3].

Although autodisplay platforms provide many attractive features for the CSD system, constraints still exist that limit the versatility of displayed proteins [4]. One such constraint is the retention of the functionality of proteins displayed in a multimeric form. A few reports have described the functional display of proteins having a dimeric active form by spontaneous dimerization within the limited space of the cell surface [5, 6]. Because spontaneous dimerization can only occur between monomers in close proximity, this strategy can only be used when displayed proteins are expressed in high numbers, rendering this approach highly conditional. In some cases, there is a possibility of displayed proteins acquiring an inactive conformation due to improper folding [7].

To develop an alternative approach that can overcome these limitations, we designed a cell surface attachment (CSA) system in which POIs can be non-covalently attached to surface-displayed anchoring scaffolds. For non-covalent attachment, we searched protein families to select interacting protein domains that show specific binding interactions. We selected the cohesin-dockerin (Coh-Doc) domain pair to facilitate cell surface anchoring. The Coh–Doc system was originally discovered in many cellulolytic anaerobic bacteria where it is responsible for building extracellular macromolecular complexes such as the “cellulosome.” The Coh–Doc system controls and maximizes the degradation efficiency of recalcitrant cellulose using a limited amount of enzymes [8]. The interaction between the Coh and Doc domains exhibits a very high binding specificity and strong affinity in a Ca^2+^-dependent manner [9, 10]. Because Coh–Doc binding is known to be one of the strongest protein–protein interactions (Kd, 10^−9^–10^−11^) [11-14], the system has high potential as a biorecognition module [15].

We applied the Coh and Doc modules to the autodisplay platform and designed a CSA system to allow the functional expression of multi-complex proteins by non-covalent anchoring of heterologous proteins. To confer high orthogonality of applied modules, we surveyed potential Coh–Doc pairs predicted in various bacteria and selected the Coh–Doc pair from *Archaeoglobus fulgidus* [16]. Functional attachment was validated by a known dimeric enzyme (α-neoagarobiose hydrolase, NABH) that had previously failed to display functionally in the autodisplay platform [17]. In this study, we constructed a novel CSA system using Coh–Doc pairs as biorecognition modules and provided a promising tool for the extension of autodisplay platform applications.

## Materials and Methods

### Bacterial Strains, Plasmids, and Culture Conditions

*Escherichia coli* DH5α and *E. coli* BL21(DE3) were used as host strains for general cloning and recombinant protein overexpression, respectively. *E. coli* BW25113 was used for surface display of the Coh or Doc module. All *E. coli* cultures were aerobically grown in Luria–Bertani (LB) medium (Difco, USA) supplemented with 100 μg/ml ampicillin as the selectable marker. All *E. coli* competent cells were prepared by the TSS method [18]. *Saccharophagus degradans* 2-40^T^ (ATCC 43961) was cultivated in a sea salt minimal medium as described by Ekborg *et al*. [19]. The genes encoding the Coh (Accession No. O30294) and Doc (Accession No. O30295) domains of *A. fulgidus* DSM 4304 were synthesized and codon optimized for *E. coli* from GeneArt (hereafter referred to as AfuCoh76 and AfuDoc75, respectively). The bacterial strains, plasmids, and primers for PCR are listed in Table 1.

### Construction and Cloning of Monomeric Red Fluorescent Protein (mRFP1) and NABH Tagged with AfuCoh76 and AfuDoc75 Modules

The gene sequences of AfuCoh76, AfuDoc75, mRFP1 [20], and NABH [21] were amplified by PCR using α-Taq polymerase (GeneAll, Korea). To fuse mRFP1 to AfuCoh76 and AfuDoc75, overlap extension PCR (OE-PCR) was performed [22]. For the overexpression of fusion proteins, the resulting PCR products were cloned into a pJL vector having a 6xHis-tag at the C-terminus for affinity chromatography purification (pJL-mRFP1:AfuCoh76, -mRFP1:AfuDoc75, -NABH:AfuDoc75, and -AfuDoc75:NABH; the position of AfuCoh76 and AfuDoc75 in the fusion proteins indicates the location at either the C- or the N-terminus) [23]. To autodisplay AfuCoh76 and AfuDoc75, the resulting PCR products were cloned into a pATLIC vector (pATLIC-AfuCoh76 and -AfuDoc75)[17]. All recombinant clones were confirmed by DNA sequencing.

### Overexpression and Purification of mRFP1:AfuCoh76 and mRFP1:AfuDoc75

To purify mRFP1 tagged with either AfuCoh76 or AfuDoc75, *E. coli* BL21(DE3) cells having the designated plasmids were grown in 100 ml of LB media with ampicillin (100 μg/ml) at 37°C and 200 rpm to an OD_600_ of 0.8 and added to a final concentration of 0.5 mM isopropyl thio-β-D-galactoside, and the cells were induced at 37°C for 6 h. The cells were harvested by centrifugation at 5,000 ×*g* at 4°C for 30 min, resuspended in 0.1 M Tris-HCl buffer (pH 8.0), and disrupted by sonication at 4°C for 15 min. Crude cell extracts were centrifuged at 15,000 ×*g* (at 4°C for 50 min) to remove the cell debris. The resulting supernatant solution was placed on a histidine affinity column (HiTrap HP, GE Healthcare, USA) equilibrated with a 20 mM Tris-Cl buffer (pH 8.0) in an LP system (Bio-Rad, USA). The rate of sample loading and column elution was maintained at 3.0 ml/min by the LP system. The recombinant proteins were eluted with a linear gradient of imidazole (0–0.5 M) included in the same buffer, and active fractions were collected. Combined fractions were concentrated with Amicon Ultra-15 Centrifugal Filter Units (10,000 NMWL) (3,000 ×*g* at 4°C for 1–2 h) and stored at 4°C for further experimentation.

### Analysis of Non-Denaturing Polyacrylamide Gel Electrophoresis

In total, 10 μM each of purified mRFP1:AfuCoh76 and mRFP1:AfuDoc75 were combined in a 100-μl mixture of 2 mM CaCl_2_ and Tris-HCl (pH 8.0) for 30 min at room temperature for the formation of a complex between the AfuCoh76 and AfuDoc75 modules. Non-denaturing native polyacrylamide gel electrophoresis was performed using a 1.5-mm thick 10% acrylamide resolving gel prepared in 125 mM Tris-HCl (pH 8.8). Samples were prepared in 62.5 mM Tris-HCl (pH 6.8) containing 10% (w/v) glycerol and 0.01% (w/v) bromophenol blue, but without 2-mercaptoethanol and SDS. The prepared samples were loaded on the 10% acrylamide gel without heating. Electrophoresis was performed in Tris-Glycine buffer (25 mM Tris and 192 mM glycine, pH 8.8) with 2 mM CaCl_2_. Protein bands were stained for 30 min using 0.25% (w/v) Coomassie brilliant blue and destained using a solution containing 20% (v/v) methanol and 10% (v/v) acetic acid.

### Display of AfuDoc75 and AfuCoh76 on the Surface of *E. coli*

The resulting plasmids (pATLIC-AfuDoc75 and -AfuCoh76) were transformed into *E. coli* BW25113. The *E. coli* cells having the designated plasmids were cultured in 100 ml of LB media supplemented with ampicillin (100 μg/ml) at 37°C and were induced at an OD_600_ of 0.6 by L(+)-arabinose (final concentration of approximately 0.02%) at 16°C for 24 h. Displayed cells were harvested by centrifugation (3,000 ×*g* at 4°C for 10 min), and the cell pellet was washed with ice-cold 10 mM NaCl and stored at −20°C for further experimentation.

### Verification of CSA Using mRFP1 Tagged with AfuCoh76 and AfuDoc75

In total, 10 μM of purified mRFP1:AfuDoc75 and mRFP1:AfuCoh76 was mixed with 2 ml of culture (approximately 1 × 10^9^ cells) of the cells displaying AfuDoc75 and AfuCoh76 at room temperature for 30 min in the presence of 2 mM CaCl_2_. After washing the unbound mRFP1 with PBS, the surface-bound mRFP1, bound by the interaction between the AfuCoh76 and AfuDoc75 modules, was measured using a Victor3 spectrophotometer (Perkin-Elmer) with excitation at 590 nm (20 nm bandwidth) and emission at 616 nm (8.5 nm bandwidth) in a 96-well plate. The background fluorescence of the cells was subtracted to obtain the relative fluorescence units.

### Detection of Whole Cell Activity for Surface-Attached Dimeric NABH in *E. coli*

To efficiently purify proteins tagged with AfuDoc75, the crude cell extract containing overexpressed NABH (induction at 16°C for 24 h) was directly mixed with 2 ml of culture (approximately 1 × 10^9^ cells) of the cells displaying AfuCoh76. The mixture was added to a final concentration of 2 mM CaCl_2_ and incubated for 30 min at room temperature. Enzyme-displaying cells were collected by centrifugation (3,000 ×*g* at 4°C for 5 min) and incubated in 1 ml reaction mixture (2 mM CaCl_2_ and 20 mM Tris-HCl, pH 8.0) including 1.0% neoagarobiose (DP2, NAB) as the substrate of NABH at 25°C for 3 h. The reaction products were analyzed by thin-layer chromatography (TLC) in a solvent system of n-butanol:ethanol:water (3:2:2, v/v) and visualized with 10% (v/v) H_2_SO_4_ and 0.2% naphthoresorcinol in ethanol by heating [24].

## Results

### Selection of Coh–Doc Pairs Among Various Bacteria

Among 154 Coh–Doc pairs, we selected orthogonal Coh–Doc pairs that maintained specific binding between various POIs and anchoring scaffolds and that prevented cross-binding among Coh–Doc pairs. We evaluated the specificity and strength of bacterial Coh–Doc pairs based on the protein blot array image analysis [25] of the Coh–Doc pairs in the results reported by Haimovitz *et al*. [26].

Using all-against-all pairwise comparison, we identified the Coh–Doc pair of *A. fulgidus* as having the highest specificity and the strongest binding pair. The AfuCoh76-AfuDoc75 pair has previously been reported to exhibit strong and specific binding interactions [16]. Therefore, we chose the AfuCoh76-AfuDoc75 pair for the CSA system. The domain boundary was determined as previously reported [Doc domain in ORF2375 (432–506 amino acids) and Coh domain in ORF2376 (29–162 amino acids)] [16]. For heterologous expression of archaeal genes in *E. coli*, we optimized codons and synthesized AfuCoh76 and AfuDoc75 genes.

### Validation of In Vitro Binding Between mRFP1:AfuCoh76 and mRFP1:AfuDoc75 Modules

**Fig. 1:**
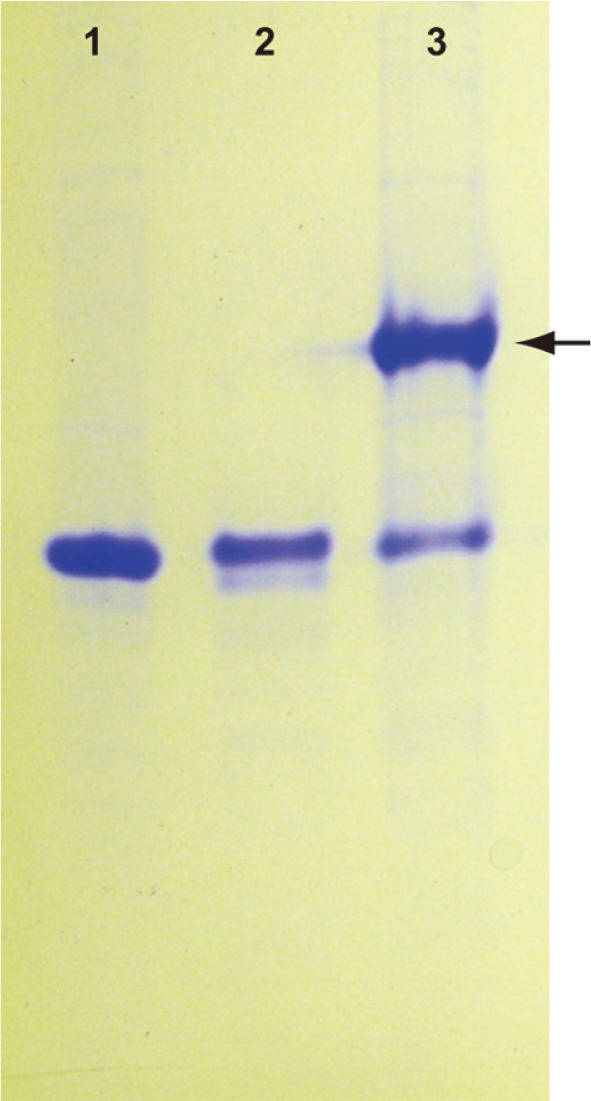
Non-denaturing polyacrylamide gel electrophoresis analysis for the confirmation of complex formation of purified mRFP1:AfuCoh76 and mRFP1:AfuDoc75. Lane 1, purified mRFP1:AfuCoh76 (approximate M.W.: 39kDa, 20 μg); lane 2, purified mRFP1:AfuDoc75 (approximate M.W.: 33.4kDa, 20 μg); lane 3, the complex in a mixture of mRFP1:AfuCoh76 and mRFP1:AfuDoc75 (arrow).

Fusion proteins are generated by linking two proteins or domains of proteins by a peptide linker. The selection of a suitable linker sequence is of particular importance in the construction of functional fusion proteins [27]. Several studies related to recombinant Doc-containing proteins have not used any other special linker sequence because there are Doc domains linked to cellulases found in nature, which have a simple structure [11, 28]. This feature offers an additional advantage when designing fusion proteins using a Doc domain as a tag. We confirmed the interaction between purified mRFP1 tagged with the AfuCoh76 or AfuDoc75 modules (mRFP1:AfuCoh76 or mRFP1:AfuDoc75, respectively) in vitro under non-denaturing conditions in the presence of Ca^2+^, which structurally maintains EF-hand coordination in the Coh and Doc modules (Fig. 1) [8]. This result indicates that each part can independently form a functional fold in fusion proteins without linker regions between heterologous proteins and the AfuDoc75 module.

### Attachment of Heterologous Proteins on the Surface of *E. coli*

**Fig. 2:**
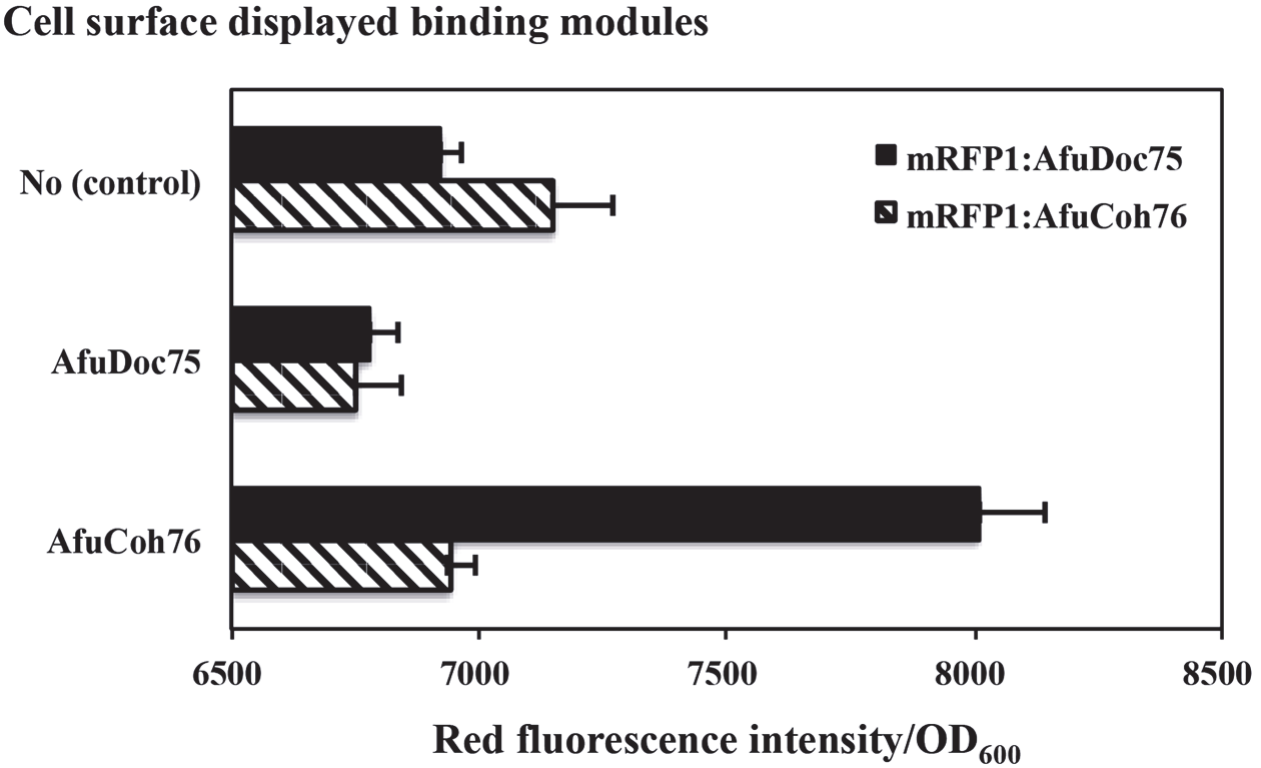
Combinatorial binding tests of mRFP1:AfuCoh76 and mRFP1:AfuDoc75 to displayed AfuCoh76 and AfuDoc75. Only mRFP1:AfuDoc75 could be anchored to the surface-displayed AfuCoh76.

To verify functional attachment on the surface of *E. coli*, we combinatorially bound purified mRFP1:AfuCoh76 and mRFP1:AfuDoc75 to AfuCoh76- and AfuDoc75-displayed *E. coli* cells, respectively. As shown in Fig. 2, we only observed functional binding in the pairing between mRFP1:AfuDoc75 and AfuCoh76-diplayed *E. coli* cells. However, we failed to observe the red fluorescence of mRFP1:AfuCoh76 on the *E. coli* cells displaying AfuDoc75 for unknown reasons. We calculated the number of copies of mRFP1:AfuDoc75 bound to the cell surface displaying AfuCoh76. Approximately 60,000 copies of mRFP1:AfuDoc75 were docked to the surface of cells, and this value corresponded to the number of copies of displayed mRFP1 reported in our previous study [17].

### CSA of NABH to the Surface of *E. coli*

One of the current challenges in the surface display system is the expression of large multi-complex proteins [29]. Although several studies have shown that proteins with dimeric forms, such as bovine adrenodoxin and sorbitol dehydrogenase, can be actively displayed on the surface of *E. coli*, these studies were performed using an autodisplay platform with a high expression rate (*e.g.*, AIDA-I autotransporter unit) [5, 6]. The results from these studies did not completely overcome the structural limitation for the dimeric conformation but only partially overcame it. Because the established autodisplay platforms based on the type V secretion system are monomeric autotransporters [30], it is impossible to display multi-complex proteins using the currently available autodisplay platforms.

In a recent structural study on NABH, the overall fold structure of the enzyme was found to be organized as an N-terminal helical extension and a C-terminal, five-bladed β-propeller catalytic domain [21]. Because the C-terminus of NABH is the structurally critical position for dimerization in the active conformation, there is no CSD system currently available that can functionally display NABH.

**Fig. 3:**
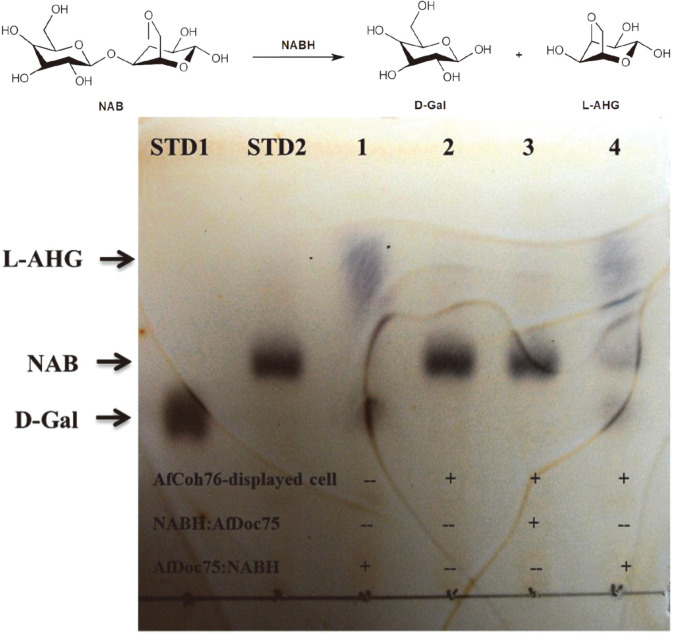
Thin-layer chromatography analysis for the activity test of AfuDoc75:NABH-anchored cells. Lane STD1, D-galactose (D-Gal); STD2, neoagarobiose (NAB); 1, the reaction products in crude extract from overexpressed AfuDoc75:NABH (positive control); 2, the reaction products from cells without displayed AfuCoh76; 3, the reaction products from NABH:AfuDoc75-anchored cells (C-terminal fusion state); 4, the reaction products from AfuDoc75:NABH-anchored cells (N-terminal fusion state). D-Gal and 3,6-anhydro-L-galactose (L-AHG) are the reaction products degraded from NAB.

For the functional cell surface attachment of NABH on the surface of *E. coli* displaying AfuCoh76, we fused AfuDoc75 to the N-terminus of NABH (designated as AfuDoc75:NABH). The purified AfuDoc75:NABH was attached to the cell surface of *E. coli* displaying AfuCoh76. As expected, based on the attachment of the active dimeric form of NABH to the cell surface, we only detected the activity of NABH in a whole cell line anchoring AfuDoc75:NABH by a TLC chromatogram, and there was no NABH activity in any of the other whole cell lines (Fig. 3). This result indicates that the designed CSA system has the potential to attach multi-complex proteins using non-covalent interactions between Coh and Doc modules.

## Discussion

A novel CSA system was designed using a non-covalent interaction to overcome the intrinsic limitations of the autodisplay platforms currently available. In the designed CSA system, it is possible to display multi-complex proteins, and there is no concern that disulfide bonds in displayed heterologous proteins may affect successful translocation to the outer membrane [4].

Although there are several modules present on bacterial cell surfaces that can mediate attachment via covalent or non-covalent interactions, such as S-layer homology domain [31], sortase-catalyzed cell wall attachment at LPXTG motif [32], choline-binding module [33], leucine-rich repeats [34], and lipoproteins [35], the designed CSA system that uses interactions between Coh and Doc domains offers several benefits. Because the AfuCoh76 and AfuDoc75 modules from *A. fulgidus*, in particular, exhibit the strongest recognition affinity, cross-linking to other cellular components can be effectively prevented. This system has the ability to tag the Doc module to the N-or C-terminus of anchored proteins without any linker sequence. It also has the potential for reversible specific binding by a Ca^2+^ switch by modifying the Ca^2+^-binding loop of the Doc module [36]. This system also offers the ability to directly decorate the Coh-displayed cells from crude cell extracts expressing Doc-fused proteins in a single step followed by simple purification steps.

In our previous study, although we failed to functionally display NABH through the YfaL autodisplay platform [17], we successfully and functionally anchored it to the surface of *E. coli* using AfuCoh76 and AfuDoc75 as biorecognition modules. The combination of the autodisplay platform and Coh–Doc module provides the opportunity to functionally display various multi-complex proteins on the surface of gram-negative bacteria.

## Figures and Tables

**Table 1:** Bacterial strains, plasmids, and oligonucleotide primers used in this study.

Strain, plasmid, or oligonucleotide	Relevant characteristic(s), description, or sequence^a^	Source or reference
Strains		
*E. coli* DH5α	F^-^ *endA1 glnV44 thi-1 recA1 relA1 gyrA96 deoR nupG* *Φ80dlacZΔM15 Δ(lacZYA-argF)U169 hsdR17(r_K_^-^ m_K_^+^) λ–*	Invitrogen
*E. coli* BW25113	F- *Δ(araD-araB)567 ΔlacZ4787(::rrnB-3) λ^-^ rph-1 Δ(rhaD-rhaB)568 hsdR514*	CGSC^a^
*E. coli* BL21(DE3)	F– *omp*T *hsd*SB (rB–, mB–) *gal* *dcm* (DE3)	Invitrogen
*S. degradans* 2-40	Source of agarase genes and NABH	ATCC^b^
Plasmids		
AfuCoh76	The plasmid carrying *AfuCoh76* gene synthesized using GeneArt®	This study
AfuDoc75	The plasmid carrying *AfuDoc75* gene synthesized using GeneArt®	This study
pJL vector	Derivative of pET21a vector; insertion of LIC sequence	(Lee and Kim, 2009)
pJL-mRFP1: AfuCoh76	pJL carrying the fusion gene comprised AfuCoh76 fused to the C-terminus of mRFP1	This study
pJL-mRFP1: AfuDoc75	pJL carrying the fusion gene comprised AfuDoc75 fused to the C-terminus of mRFP1	This study
pJL-NABH: AfuDoc75	pJL carrying the fusion gene comprised AfuDoc75 fused to the C-terminus of NABH	This study
pJL-AfuDoc75: NABH	pJL carrying the fusion gene comprised AfuDoc75 fused to the N-terminus of NABH	This study
pATLIC vector	Autodisplay vector based on the YfaL autotransporter	(Ko *et al*. 2012)
pATLIC-AfuCoh76	pATLIC carrying the *AfuCoh76* gene	This study
pATLIC-AfuDoc75	pATLIC carrying the *AfuDoc75* gene	This study
Primers		
AfCoh76-mRFP1_F	GTCGTCACTCCACCGGTGCTGCTAGTGCTGAAATGGTGGTTA	This study
AfCoh76-pJL_R	ATGATGGTGATGGTGACCAGCACCACCTTTTACGG	This study
AfDoc75-mRFP1_F	GTCGTCACTCCACCGGTGCTGAAGAAGCGAACAAAGGCGAC	This study
AfDoc75-pJL_R	ATGATGGTGATGGTGAGGCCTTTTGCCCAGCAGGCCATTCT	This study
mRFP1_F	GAAGGAGATATAAGGATGGCTTCCTCCGAAGACGTTATC	This study
mRFP1_R	ATGATGGTGATGGTGAGCACCGGTGGAGTGACG	This study
mRFP1-AfCoh76_R	ACCACCATTTCAGCACTAGCAGCACCGGTGGAGTGACG	This study
mRFP1-AfDoc75_R	TCGCCTTTGTTCGCTTCTTCAGCACCGGTGGAGTGACG	This study
AfCoh76at_F	CGGTGTCGCGCCCGCTAGTGCTGAAATGGTGGTTA	This study
AfCoh76at_R	CGGTCGTTGGCCCACCAGCACCACCTTTTACGG	This study
pATLIC_29__R	GTTGGCCCGGGCGCGACACCGTTAGCAGAGAAAA	This study
AfDoc75at_F	CGGTGTCGCGCCCGAAGAAGCGAACAAAGGCGAC	This study
AfDoc75at_R	CGGTCGTTGGCCCTTTGCCCAGCAGGCCATTCT	This study
AfDoc75-pJL_F	GAAGGAGATATAAGGATGGAAGAAGCGAACAAAGGCGAC	This study
AFDoc75-NABH_R	TTATTTACTTTTGAATCGCTTTTGCCCAGCAGGCCATTCTGAG	This study
NABH-AfDoc75_F	AGAATGGCCTGCTGGGCAAAAGCGATTCAAAAGTAAATAAAAAATTGAG	This study
NABH-pJL_R	ATGATGGTGATGGTGTACTGCTCCGGAATCGCCTGTTTG	This study

^a^CGSC: Coli Genetic Stock Center. ^b^ATCC: American Type Culture Collection
